# Probiotic Properties of *Lactobacillus* Strains Isolated from Tibetan Kefir Grains

**DOI:** 10.1371/journal.pone.0069868

**Published:** 2013-07-22

**Authors:** Yongchen Zheng, Yingli Lu, Jinfeng Wang, Longfei Yang, Chenyu Pan, Ying Huang

**Affiliations:** Central Research Laboratory, Second Hospital of Jilin University, Changchun, People’s Republic of China; Clermont Université, France

## Abstract

The objective of this study was to evaluate the functional properties of lactic acid bacteria (LAB) isolated from Tibetan kefir grains. Three *Lactobacillus* isolates identified as *Lactobacillus acidophilus* LA15, *Lactobacillus plantarum* B23 and *Lactobacillus kefiri* D17 that showed resistance to acid and bile salts were selected for further evaluation of their probiotic properties. The 3 selected strains expressed high *in vitro* adherence to Caco-2 cells. They were sensitive to gentamicin, erythromycin and chloramphenicol and resistant to vancomycin with MIC values of 26 µg/ml. All 3 strains showed potential bile salt hydrolase (BSH) activity, cholesterol assimilation and cholesterol co-precipitation ability. Additionally, the potential effect of these strains on plasma cholesterol levels was evaluated in Sprague-Dawley (SD) rats. Rats in 4 treatment groups were fed the following experimental diets for 4 weeks: a high-cholesterol diet, a high-cholesterol diet plus LA15, a high-cholesterol diet plus B23 or a high-cholesterol diet plus D17. The total cholesterol, triglyceride and low-density lipoprotein cholesterol levels in the serum were significantly (*P*<0.05) decreased in the LAB-treated rats compared with rats fed a high-cholesterol diet without LAB supplementation. The high-density lipoprotein cholesterol levels in groups B23 and D17 were significantly (*P*<0.05) higher than those in the control and LA15 groups. Additionally, both fecal cholesterol and bile acid levels were significantly (*P*<0.05) increased after LAB administration. Fecal lactobacilli counts were significantly (*P*<0.05) higher in the LAB treatment groups than in the control groups. Furthermore, the 3 strains were detected in the rat small intestine, colon and feces during the feeding trial. The bacteria levels remained high even after the LAB administration had been stopped for 2 weeks. These results suggest that these strains may be used in the future as probiotic starter cultures for manufacturing novel fermented foods.

## Introduction

Tibetan kefir grains are the natural starter for fermented milk in Tibet, China. The milk fermented by Tibetan kefir grains is a type of self-carbonated yogurt called Tibetan kefir, which is a traditional food of Tibetan people. Statistical data show that people who consume kefir in their diet are longevous. The data suggest that probiotic bacteria in the gut of kefir consumers are abundant and diverse, and microbial communities in the gut are closely correlated with health [1], [2]. Kefir has been assigned a variety of health claims in addition to its nutritional value [3]. Many studies regarding kefir’s biological activities have established that kefir has anti-inflammatory activity, immune-modulating activity, antimicrobial activity and anti-proliferative activity, and it has the potential to become a type of functional food [4]–[7]. Tibetan kefir grains contain a complex microbial community composed of lactic acid bacteria (LAB) (*Lactobacillus*, *Lactococcus* and *Leuconostoc*) and yeasts (*Saccharomyces*, *Kluyveromyces* and *Torula*) [8]. Various LAB that have been isolated from Tibetan kefir grains include *Lactobacillus acidophilus* [9], *Lactobacillus plantarum* [10] and *Lactobacillus kefiranofaciens* [11].

Research into novel probiotic strains is important to satisfy the increasing market demand and to obtain highly active probiotic cultures for improved products [12] with probiotic characteristics that are superior to those presently on the market.

The objective of this study was to identify the probiotic properties of *Lactobacillus* strains isolated from Tibetan kefir grains. The isolates were preliminarily selected based on acid and bile tolerance, and the selected isolates were further screened for various functional properties, such as adhesion ability, antibiotic resistance and cholesterol-lowering activity. Three strains showing desirable properties were selected for further assay of their cholesterol-lowering effect in rats, and the survival of the strains after passage through rat intestines was also examined in 4-week feeding trials.

## Results

### Acid and Bile Tolerance

In total, 10 Gram-positive, catalase-negative, rod-shaped isolates were obtained from kefir grains. Table 1 shows the survival of the 10 isolates under low pH and bile salt conditions. All of the strains except for C02 consistently showed tolerance at pH 3, and the residual counts were greater than 10^6^ CFU/ml after 3 h of incubation. Only 7 isolates survived at pH 2, and the LA15, B23 and D17 isolates exhibited fairly high levels of acid tolerance, with maintenance levels greater than 85% after exposure to pH 2. In the bile salts test, all of the tested isolates were able to grow in 0.3% bile, but only LA15, B23 and D17 showed resistance at a concentration of 1% bile for up to 12 h. The isolates LA15 and B23 showed comparatively better tolerance and yielded viable counts greater than 10^7^ CFU/ml in 1% bile. Based on the screening results for tolerance to low pH and bile salts, 3 isolates, LA15, B23 and D17, were able to survive at levels of 10^6^ CFU/ml under pH 2 or 1% bile salts conditions and were therefore selected for further analysis of their probiotic properties.

**Table 1:** Acid and bile tolerance of LAB strains (log CFU/ml).

Isolate name	Initial mean counts^a^	Resistant to gastric juice		Bile tolerance		
		pH 2	pH 3	0.3%	0.5%	1%
A06	8.98±0.12	2.30±0.14	6.34±0.23	3.71±0.32	2.31±0.25	–
A18	9.03±0.16	–b	6.28±0.15	3.25±0.12	–	–
LA03	8.97±0.11	–	6.52±0.23	4.67±0.31	3.72±0.22	–
LA15	9.10±0.17	8.23±0.24	8.72±0.21	7.76±0.24	7.64±0.26	7.58±0.23
B05	9.02±0.18	–	6.53±0.24	3.28±0.16	–	–
B07	8.97±0.15	3.12±0.19	6.49±0.16	3.45±0.13	2.17±0.13	–
B23	8.99±0.23	7.76±0.17	8.68±0.26	7.49±0.20	7.71±0.22	7.84±0.33
C02	9.01±0.19	–	–	3.63±0.26	–	–
C09	9.03±0.14	4.57±0.13	6.73±0.14	4.12±0.23	3.59±0.18	–
D17	8.97±0.21	7.74±0.15	8.59±0.18	6.37±0.23	6.64±0.18	6.82±0.21

a Each value represents the mean value ± standard deviation (SD) from the 3 trials.

b No growth.

### Identification of Lactic acid Bacteria

The LA15, B23 and D17 isolates were identified as *Lactobacillus acidophilus*, *Lactobacillus plantarum* and *Lactobacillus kefiri*, respectively, using the API 50CHL test. The 16S rDNA of each of the 3 isolates was sequenced, and the organisms were identified by alignment of these 16S sequences as *Lactobacillus acidophilus* LA15 (Genbank accession no. KC166236), *Lactobacillus plantarum* B23 (Genbank accession no. KC166237) and *Lactobacillus kefiri* D17 (Genbank accession no. KC155629).

### Adhesion Properties

**Figure 1:**
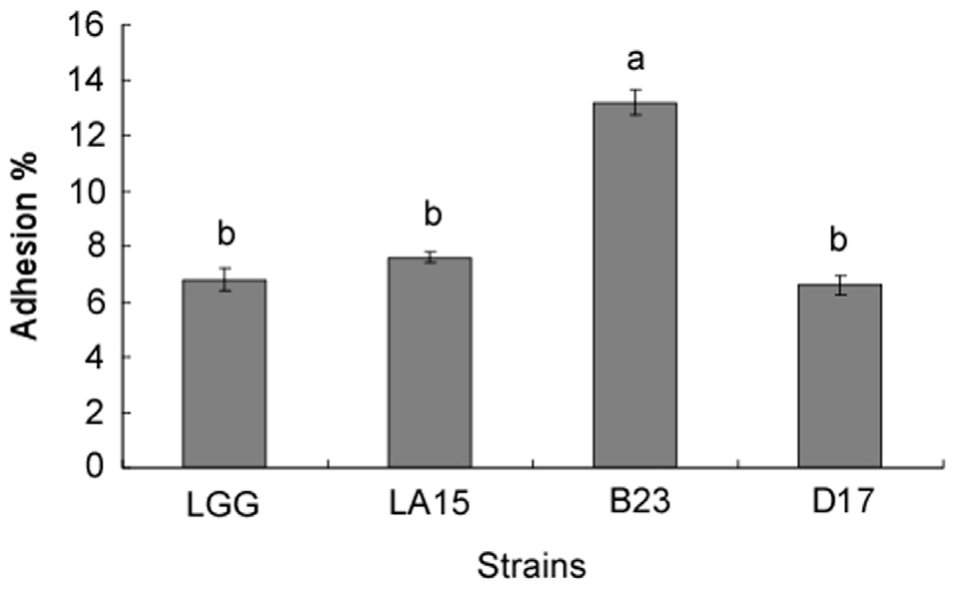
Adhesion ability of *Lactobacillus* isolates to Caco-2 epithelial cells compared with the reference strain *Lactobacillus rhamnosus* GG (LGG). The presented values are the means of triplicate determination, and the different letters (a, b) represent significant differences (*P*<0.05).

The adhesion rates to Caco-2 cells varied depending on the tested strains and ranged from 6.5 to 13.2% (Fig. 1). B23 showed significantly better binding rates than the reference strain LGG. The adhesion rates of strains LA15 and D17 were significantly lower (*P*<0.05) than those of strain B23. The binding abilities of LA15 and D17 were equivalent to the binding ability of LGG.

### Antibiotic Susceptibility Testing

All 3 strains were found to be susceptible to gentamicin, erythromycin and chloramphenicol but were resistant to vancomycin, with MIC values of 26 µg/ml. LA15, which was also resistant to tetracycline, with an MIC value of 45 µg/ml, was tested by PCR for the presence of *tet* resistance genes. After PCR amplification, only *tet* (M) gene was detected in strain LA15 (data not shown).

### Cholesterol Lowering Effects

All selected strains showed bile salt deconjugation ability using the BSH test on agar plates (Table 2). The variance between the abilities of the 4 strains to assimilate cholesterol was significant (*P*<0.05). The amounts of cholesterol assimilated ranged from 62.63 to 56.19 µg/ml. Strains B23 and D17 exhibited significantly higher amounts (*P*<0.05) of cholesterol assimilation compared with LA15 and LGG. Cholesterol was co-precipitated concurrently with deconjugated sodium glycocholate at varying levels (*P*<0.05), ranging from 2.36 to 3.89 µg/ml in the 4 cultures. Deconjugation of sodium glycocholate by B23 and D17 resulted in a significantly higher amount (*P*<0.05) of cholesterol co-precipitation compared with LA15.

**Table 2:** *In vitro* test of cholesterol-related activities of LAB isolated from Tibetan kefir grains.

Strains	Bile salt hydrolase activity	Cholesterol assimilation(µg/ml)	Cholesterol co-precipitation(µg/ml)
LA15	+	56.19±0.53^b^	2.36±0.11^b^
B23	+	62.63±0.41^a^	3.89±0.13^a^
D17	+	61.58±0.62^a^	3.52±0.24^a^
LGG	+	55.32±0.46^b^	3.74±0.18^a^

a, b Mean values within the same column with different superscripted letters differ significantly (*P*<0.05).

The data are shown as the mean ± standard deviation.

### Food Intake, Weight Gain and Food Efficiency

All rats were generally healthy throughout the feeding trial period. The 4 groups of rats showed no significant (*P*>0.05) differences in body weight gain, food intake or food efficiency. This indicates that the animals supplemented with LAB strains grew in similar patterns compared with the control (Table 3).

**Table 3:** Body weight gain, total food intake and feeding efficiency of rats fed a high-cholesterol diet (control) or supplemented with different lactic acid bacteria strain (LA15, B23 and D17) diets after 4 weeks.

		Treatment groups		
Item	Control	LA15	B23	D17
Body weightgain (g)	171.7±8.9^a^	173.6±6.9^a^	174.4±9.5^a^	169.3±7.2^a^
Total foodintake (g)	468.6±11.5^a^	470.9±8.7^a^	472.8±8.7^a^	469.9±6.8^a^
Food efficiency* (%)	36.6±2.1^a^	36.9±2.3^a^	36.9±2.6^a^	36.0±1.8^a^

a Mean values within a row with different superscripted letters differ significantly (*P*<0.05).

The data are shown as the mean ± standard deviation, n = 10.

* Food efficiency (%) = (body weight gain/food intake) ×100.

### Blood Lipid Analysis

**Figure 2:**
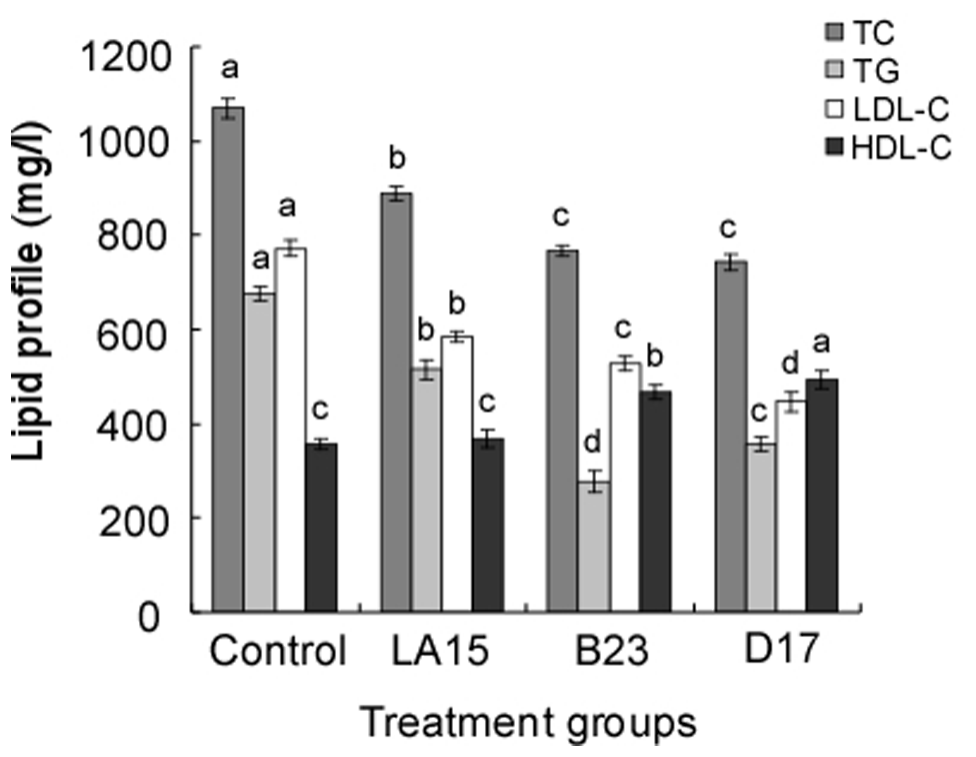
Total cholesterol (TC), triglycerides (TG), high-density lipoprotein cholesterol (HDL-C) and low-density lipoprotein cholesterol (LDL-C) levels in the serum of rats fed a high-cholesterol diet alone (control) or supplemented with different lactic acid bacteria strains (LA15, B23 or D17) for 4 weeks. The results are expressed as the means ± standard deviation, n = 10. Means within the same lipid series with different lowercase letters (a-d) are significantly different (*P*<0.05).

The serum TC, TG, HDL-C and LDL-C levels in the 4 groups are shown in Figure 2. Rats fed the LA15, B23 and D17 diets had significantly (*P*<0.05) lower TC levels compared with the control group. Groups B23 and D17 had significantly (*P*<0.05) lower TC levels than group LA15. Groups B23, D17 and LA15 showed a significant (*P*<0.05) reduction in TG levels compared with the control group. Group B23 had the lowest TG levels. Group D17 expressed the lowest LDL-C levels, followed by group B23 and the LA15 and control groups. The differences in LDL-C levels between the 4 groups were significant (*P*<0.05). The HDL-C levels in groups B23 and D17 were significantly (*P*<0.05) higher than in the control and LA15 groups.

### Liver and Fecal Cholesterol

Table 4 shows the liver and fecal cholesterol levels and the weights of the livers. The average liver weight was not significantly different between the groups (*P*>0.05). Groups LA15, B23 and D17 had a significantly reduced liver cholesterol content compared with the control group (*P*<0.05). The differences between the LAB-treated groups were not significant (*P*>0.05). The fecal cholesterol concentration of rats fed the LAB strains significantly increased compared with the control group (*P*<0.05). Groups B23 and D17 showed significantly higher fecal cholesterol concentrations than group LA15.

**Table 4:** The liver weight and total cholesterol levels in liver and feces of rats fed a high-lipid diet alone (control) or supplemented with different lactic acid bacteria strains (LA15, B23 or D17) for 4 weeks.

		Treatment groups		
Item	Control	LA15	B23	D17
Liverweight (g)	9.05±0.06^a^	9.03±0.09^a^	9.06±0.08^a^	9.04±0.11^a^
Liver cholesterol (mg/g)	16.88±0.18^a^	11.25±0.09^b^	10.95±0.12^b^	12.05±0.08^b^
Fecal cholesterol (mg/g)	10.68±0.15^b^	15.93±0.19^a^	16.13±0.21^a^	15.88±0.25^a^

a–c Means within the same row followed by different superscript letters are significantly different (*P*<0.05).

The data are shown as the mean ± standard deviation.

### Fecal Total Bile Acid Excretions

**Figure 3:**
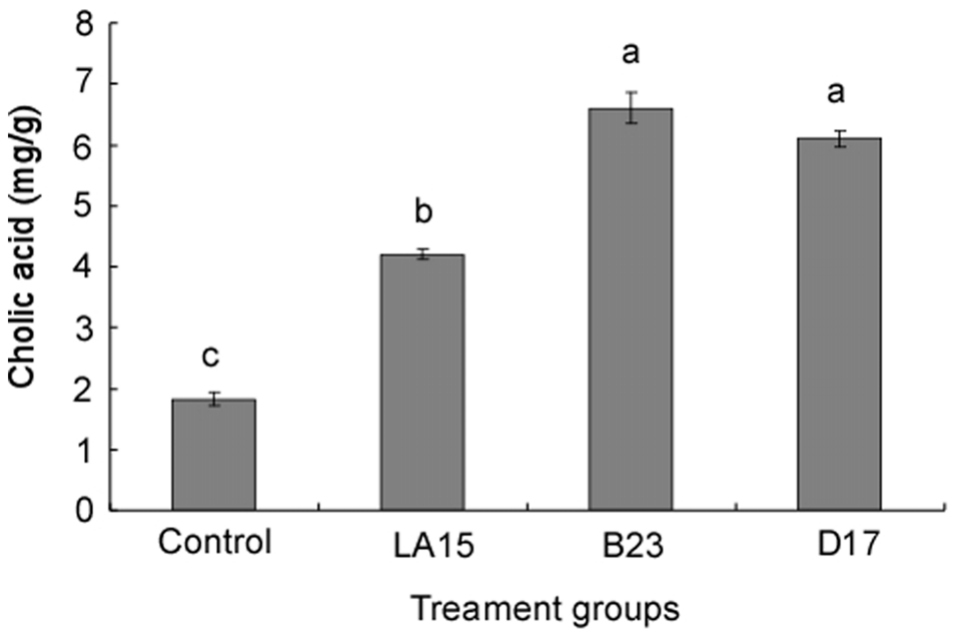
Fecal cholic acid concentrations in rats fed a high-cholesterol diet alone (control) or supplemented with different lactic acid bacteria strains (LA15, B23 or D17) for 4 weeks. The results are expressed as the mean ± standard deviation. Mean values with different letters (a, b, c) differ significantly (*P*<0.05).

Figure 3 shows the fecal total bile acid excretions in rats. The fecal total bile acid excretion was significantly (*P*<0.05) higher in rats fed the B23 and D17 diets than in the other groups. The fecal total bile acid excretion was similar among the control and LA15 groups.

### Colonization by LAB Strains (LA15, B23 or D17) in the Rat Intestinal Tract

As shown in Table 5, LA15, B23 or D17 were not detected in the duodenum, jejunum, ileum, colon and feces of rats at day 0. However, they were detected respectively in the jejunum, ileum, colon and feces after 28-day gavage, except in the duodenum. Counts of the 3 strains were significantly (*P*<0.05) higher in the ileum, colon and feces than those in the jejunum. At day 42, after stopping bacteria administration for 2 weeks, the 3 strains were still detected in the small intestine, colon and feces. The numbers of the bacteria had slightly declined.

**Table 5:** Counts of different lactic acid bacteria strains (LA15, B23 or D17) in the duodemun, jejunum, ileum, colon and feces of rats.

Treatment groups	Days	Duodenum	Jejunum	Ileum	Colon	Feces
LA15	0	0	0	0	0	0
	28	0	2.43±0.02^c^	3.63±0.03^b^	5.19±0.04^a^	5.12±0.04^a^
	42	0	2.36±0.02^c^	3.38±0.02^b^	5.07±0.03^a^	4.98±0.02^a^
B23	0	0	0	0	0	0
	28	0	2.78±0.03^c^	3.89±0.02^b^	5.53±0.03^a^	5.23±0.02^a^
	42	0	2.53±0.02^c^	3.39±0.03^b^	5.14±0.02^a^	5.08±0.03^a^
D17	0	0	0	0	0	0
	28	0	2.67±0.02^c^	3.59±0.02^b^	5.13±0.03^a^	5.07±0.04^a^
	42	0	2.47±0.03^c^	3.21±0.03^b^	4.57±0.02^a^	4.81±0.03^a^

a, b, c Mean values within a column with different superscript letters were significantly different (*P*<0.05).

The data are shown as the mean ± standard deviation.

### Microbial Analysis

Table 6 summarizes the microbial population composition of the fecal samples of the treated and control groups, as determined by colony counts on selective media. The total anaerobe counts were significantly higher in each LAB-treated group than the control group. We observed a significant increase in the number of fecal lactobacilli in the treated rats compared to that with the control rats for each tested isolate. Conversely, for each LAB-treated group, the counts of coliform organisms in the rat feces significantly decreased at day 28. After 28 days of administration, the counts of coliform organisms were remained stable until the end of 42 days.

**Table 6:** Effects of probiotic feeding on fecal bacterial population.

Treatment groups	Days	Total anaerobes	*Lactobacillus*	Coliforms
Control	0	9.24±0.02^b^	8.99±0.04^b^	6.85±0.02^a^
	28	9.21±0.03^b^	9.04±0.03^b^	6.89±0.03^a^
	42	9.25±0.03^b^	9.05±0.04^b^	6.93±0.02^a^
LA15	0	9.23±0.05^b^	9.02±0.03^b^	6.88±0.03^a^
	28	10.38±0.04^a^	9.56±0.03^a^	4.59±0.02^b^
	42	9.90±0.03^a^	9.36±0.02^a^	4.62±0.02^b^
B23	0	9.19±0.03^b^	9.04±0.03^b^	6.86±0.04^a^
	28	10.28±0.04^a^	9.66±0.04^a^	4.59±0.02^b^
	42	9.84±0.03^a^	9.47±0.03^a^	4.91±0.03^b^
D17	0	9.21±0.02^b^	8.97±0.03^b^	6.91±0.03^a^
	28	10.78±0.04^a^	9.66±0.04^a^	4.92±0.04^b^
	42	9.94±0.03^a^	9.59±0.03^a^	5.15±0.03^b^

a, b Mean values within a column with different superscript letters were significantly different (*P*<0.05).

The data are shown as the mean ± standard deviation.

## Discussion

One of the required properties for probiotics is the ability to survive in the upper gastrointestinal (GI) tract. Before reaching the distal part of the intestinal tract and exerting their probiotic effect, these bacteria must survive during transit through the stomach and upper part of the intestinal tract [13]. In this study, 3 strains (LA15, B23 and D17) were resistant to pH 2 even after 3 h of exposure, but most of the isolates showed reduced viability after exposure to pH 2 conditions. *Lactobacillus plantarum* B23 grew better than *Lactobacillus plantarum* Lp27 isolated from Tibetan kefir grains in our previous study [14] at a low pH and in the presence of high bile salt concentrations (data not shown). Bile plays a fundamental role in the specific and nonspecific defense mechanisms of the gut; the magnitude of its inhibitory effects is determined primarily by the concentration of bile salts [15]. The relevant physiological concentrations of human bile range from 0.3% to 0.5% [16], [17]. There was considerable variability in resistance to bile salts between the different species of *Lactobacillus*, supporting the importance of assessing the bile tolerance of isolates in selecting potential probiotics. In this study, strains LA15, B23 and D17 were resistant to high ox gall concentrations, most likely due to the expression of bile-resistance related proteins in bacterial cells [18].

Adhesion and colonization of probiotic bacteria in the GI tract of the host is believed to be an essential feature required for the delivery of their health benefits [19]. In fact, adhesion is a prerequisite for colonization [20], stimulation of the immune system [21] and antagonistic activity against enteropathogens [22]. In our study, the adhesion abilities of B23 to Caco-2 cells were significantly stronger than that of the reference strain LGG. A15 and D17 showed a binding ability similar to LGG. Although the conclusions drawn from results of the *in vitro* studies cannot be directly applied to the *in vivo* situations, an association between adhesion ability and temporary colonization of the human intestinal tract has been previously shown [23]. Hence, the strains LA15, B23 and D17 were selected for the animal feeding trials because of their adhesive abilities.

The importance of assessing the antibiotic resistance profile pattern of new isolates is to restrict use of probiotic cultures harboring transferable antibiotic-resistance genes. In our study, the 3 selected strains were tested for susceptibility to 5 antibiotics belonging to the most clinically relevant antibiotic classes. The antibiotic susceptibility tests indicated that they were resistant to vancomycin. These results were expected, as lactobacilli are known to be naturally resistant to vancomycin; this resistance is usually intrinsic, chromosomally encoded and not transmissible [24], [25]. LA15 was also resistant to tetracycline and found to contain the *tet*(M) gene. According to EFSA [26], when a strain of a typically susceptible species is resistant to a given antimicrobial drug, it is considered to have an acquired resistance. Acquired resistance can be due either to the presence of additional genes (genes acquired by the bacteria via gain of exogenous DNA) or the mutation of indigenous genes. To determine the origin of the resistance, PCR was applied to search LA15 for the presence of genes *tet*(L), *tet*(M) and *tet*(S), which are horizontally transferred genes associated with tetracycline resistance [27]. Only *tet*(M) gene was detected in strain LA15 (data not shown).

A high concentration of cholesterol in the blood streams of humans has been recognized as a risk factor for coronary heart disease [28], [29]. Consumption of fermented milk products containing certain lactobacilli or bifidobacteria has been claimed to decrease the concentration of bloodstream cholesterol in humans [30], [31]. Some reports [32], [33] have indicated that strains of lactobacilli that were able to assimilate cholesterol *in vitro* were also able to reduce cholesterol *in vivo*. In this study, we selected 3 strains that had similar levels of *in vitro* bile-salt resistance and low pH tolerance but with variant cholesterol-assimilating properties to determine how their cholesterol metabolism would affect rats fed a high cholesterol diet. The 3 strains showed cholesterol-reducing activity in *in vitro* experiments. Thus, we attempted to characterize their cholesterol-lowering effects in rats fed a high-cholesterol diet to determine their potentials as agents for improving serum profiles. The results showed that the 3 *Lactobacillus s*trains were all effective at reducing serum TC, TG, LDL-C and liver TC compared with the control group. Groups B23 and D17 demonstrated significantly (*P*<0.05) lower cholesterol levels than group LA15. This was in agreement with the *in vitro* results in which strains B23 and D17 exhibited greater amounts of cholesterol assimilation than LA15. The present animal studies showed a significant increase in the level of HDL-C in the groups fed B23 and D17 compared with the control and LA15 groups. The results agreed with other findings [32], [34] that animals fed a diet rich in cholesterol had a reduction in total cholesterol levels and an increase in the HDL fraction when supplied with a daily intake of LAB.

LAB might influence serum cholesterol by the promotion of fecal bile acid excretion [35], [36]. We found more bile acids in the feces of the LAB-treated rats, especially in those rats in the B23 and D17 groups. Therefore, a possible mechanism for how the 3 strains lowered the cholesterol levels may be that more of the bile acids in the enterohepatic circulation were deconjugated, precipitated and excreted in the feces, resulting in reduced serum cholesterol concentrations. This may partially explain that why rats in the B23 and D17 groups had lower serum cholesterol levels than rats in the LA15 group. Additionally, more fecal cholesterol was detected in the B23 and D17 group rats. LAB may assimilate dietary cholesterol by incorporating it into their cellular membranes or cell walls prior to the fecal excretion of the bacteria [37]. Thus, LAB strains with hypocholesterolemic properties may lower serum cholesterol in multiple ways.

In order to evaluate the colonization of the selected 3 strains to gastrointestinal mucosa, analysis of the small intestine, colon and feces samples was conducted after LAB administration. Viable LA15, B23 or D17 cells were retrieved in the feces, small intestine and colon after 28 days of supplementation and maintained at a considerable level after stopping *Lactobacillus* strains administration for 2 weeks. These data indicated that the selected 3 strains successfully colonized the gut of rats and persisted efficiently in the mucosa of the small intestine and colon.

Surviving passage through the GI tract is considered to be important for probiotics to function effectively in the intestine [38]. In the present study, we specifically examined the effects of experimental diets containing LA15, B23 and D17 strains on the fecal bacterial population. A fecal microbial analysis revealed significantly higher fecal lactobacilli counts in the probiotic treatment groups compared with that of the control group. This could be attributed to their ability to survive at low pH and high bile concentration, as described for *in vitro* experiments. However, the fecal coliforms levels were significantly decreased. Our results are in agreement with those of Liong, who reported that decreased fecal coliform counts are due to probiotic feeding in rats [39]. Such potentially probiotic bacteria colonizing the intestinal mucosa provide a barrier effect against pathogens by using a variety of mechanisms, occupation of niches, competition for nutrients and production of antimicrobials [40].

In conclusion, we successfully identified 3 *Lactobacillus* strains from Tibetan kefir grains that show satisfactory properties for probiotic application. The present study indicates that the *Lactobacillus* strains analyzed here can be useful in the production of dairy foods for potential human health benefits. Further studies are required to focus on improving the technological characteristics of probiotic strains and various properties of fermented milks by optimizing the physicochemical variables commonly used at the industrial level.

## Materials and Methods

### Tibetan Kefir Grains

Kefir grains collected from Tibet, China, were evaluated. The kefir grains were activated by adding 20 g of grains to 500 ml of sterilized milk and incubating at 25°C for 24 h. The grains were retrieved by sieving and were then re-inoculated into fresh milk and incubated at 25°C for 24 h. The grains were then considered active and used throughout this study.

### Isolation of LAB

A 10 g aliquot of kefir grains was suspended in 90 g of sterile saline buffer (0.85%) and homogenized with a Stomacher (Laboratory Blender Stomacher 400, Seward, UK) for 20 min. Serial decimal dilutions were made using the homogenized suspensions of kefir grains. Each diluted solution was spread-plated onto de Man, Rogosa and Sharpe (MRS) agar (Difco, Sparks, MD, USA). The plates were incubated in an anaerobic chest with an AnaeroPack (Mitsubishi Gas Chemical Co., Inc., Tokyo, Japan) at 30°C for 48 h. The bacterial colonies that developed on the plates were individually picked and streaked onto fresh MRS agar plates by dilution streaking to obtain single colonies; these colonies were maintained on MRS agar for immediate use and stored in 20% glycerol at −80°C. The isolates were first screened for catalase activity and Gram staining, and only those that were catalase-negative and Gram-positive were selected for further studies.

### Acid Tolerance

Each cell suspension of the selected LAB cultures was prepared by pelleting cultures grown overnight (37°C) and then washing and resuspending them in peptone saline at a concentration of 10^9^ colony-forming units (CFU)/ml. MRS medium preadjusted to pH 2.0 and 3.0 was inoculated with approximately 10^8^ CFU/ml of selected LAB cultures and incubated at 37°C for 3 h. Viability was determined using the plate count method. One-milliliter samples were drawn from each tube at 0, 1, 2 and 3 h and pelleted and washed, and tenfold serial dilutions were made using peptone saline diluent. The dilutions were plated on MRS agar and incubated anaerobically at 37°C for 24–48 h.

### Bile Salt Tolerance

The bile salt solutions were prepared using ox gall (Sigma, St. Louis, MO, USA) powder at final concentrations of 0.3%, 0.5% and 1%. Sterile double-distilled water without ox gall was used as a control. All of the solutions were autoclaved, and 10 ml of each solution was transferred into sterile test tubes. Cell suspensions containing approximately 10^9 ^CFU/ml were added to each solution (i.e., 0.3%, 0.5%, 1% and control) and incubated at 37°C in the anaerobic chest. After a 12 h incubation, 1 ml of each culture was diluted in a sterile 9 ml aliquot of a 0.85% saline blank. Plates were incubated in the anaerobic chest at 37°C for 24–48 h.

### Identification and Biochemical Characterization of the Isolates

The isolates were identified using the commercially available API 50CHL system (Biomerieux S.A., La Balme les Grottes, France) and an analysis of 16S rRNA gene sequences. The nucleic acids of each isolate were extracted using a DNA purification kit (Promega, USA) according to the manufacturer’s instructions. Two universal primers, 27f and 1492r, were used for the amplification of the 16S rRNA gene [41]. Amplicons were later sequenced (Bioasia Co. Shanghai, China) and compared with those found in the National Center for Biotechnology Information (NCBI) database. The 16S sequences were then submitted to NCBI.

### In vitro Adhesion Assays

The adhesion of the isolates was assayed according to the method described by Jacobsen and coworkers [42]. Initially, 10^5^ Caco-2 cells were seeded in each well of a 6-well tissue culture plate. Dulbecco’s modified Eagle’s minimal essential medium (DMEM) supplemented with 10% (v/v) heat-inactivated (30 min at 56°C) fetal bovine serum, 100 U/ml penicillin and 100 mg/ml streptomycin was used for culturing. The medium was replaced with fresh medium every other day. The adhesion assay was performed after 20 days of post-confluence. The cells were then washed twice with 3 ml of phosphate-buffered saline (pH 7.4), and 2 ml of DMEM without serum and antibiotics was added to each well and incubated at 37°C for 30 min. Approximately 10^9^ CFU/ml of a bacterial culture was suspended in 1 ml of DMEM medium (without serum and antibiotics) and added to the different wells. The plate was incubated at 37°C for 2 h in a 5% CO_2_/95% air atmosphere. The monolayer was washed with sterile PBS, and the cells were detached by trypsinization. A 1 ml aliquot of 0.25% trypsin-EDTA solution (Sigma, St. Louis, MO, USA) was added to each well of the 6-well plate, and the plate was then incubated for 15 min at room temperature. The serial dilutions of cell suspensions were plated onto MRS agar to determine the number of adherent bacterial cells. The plates were incubated for 24–48 h at 37°C, and the colonies were counted. The number of bacterial cells initially added to each well of the 6-well plates was also counted by serial dilution and plating on MRS agar. The results of the adhesion assay were expressed as the adhesion percentage, i.e., the ratio between adherent bacteria and added bacteria per well. Caco-2 cells of the same passage were used in 3 independent experiments (n = 3) with 2 replicates in each experiment.

### Antibiotic Susceptibility Testing

The minimum inhibitory concentrations (MICs) of gentamicin, tetracycline, erythromycin, vancomycin and chloramphenicol (Sigma, St. Louis, MO, USA) were determined for each strain using the procedure as previously described [43]. According to FEEDAP [44], the MIC breakpoint values for gentamicin, tetracycline, erythromycin, vancomycin and chloramphenicol were 8 µg/ml, 8 µg/ml, 4 µg/ml, 4 µg/ml and 4 µg/ml, respectively. PCR-based detection of the genes responsible for resistance to chloramphenicol [*cat*_pIP501_], tetracycline [*tet*(L), *tet*(M), *tet*(S)], erythromycin [*erm*(B), *erm*(C)], vancomycin (*van*A and *van*B) and gentamicin [*aac(6′)*-*aph(2′)*-*Ia*] was applied to strains suspected to carry antibiotic resistance genes. Primers and protocols were those described by Maietti et al. [45].

### Cholesterol-related Activities

The cultures were screened for bile salt hydrolase (BSH) activity by spotting 10 µl of a culture grown in MRS broth onto BSH screening medium consisting of MRS agar plates supplemented with 0.5% (w/v) sodium salt of TDCA (taurodeoxycholic acid, Calbiochem, US and Canada) and 0.37 g of CaCl_2_/l [46]. The plates were incubated anaerobically in an anaerobic jar (Difco, USA) at 37°C, and the BSH activity was determined semi-quantitatively by measuring the diameter of the precipitation zones. The assay was performed in duplicate. Cholesterol assimilation *in vitro* was determined by growing cells at 37°C in MRS broth supplemented with 0.5% w/v TDCA and 0.1 g of water-soluble cholesterol/l. Following incubation, bacterial cells were harvested by centrifugation (2,000×g for 5 min), and the supernatant and un-inoculated control MRS broth were then assayed for their cholesterol content according to the method of Mathara et al. [47]. Co-precipitation of cholesterol with cholic acid was determined based on the difference between the cholesterol levels in the control (inoculated MRS broth without bile) before and after the inoculation of MRS broth supplemented with sodium glycocholate.

### Ethics Statement

This study was carried out in strict accordance with the recommendations in the Guide for the Care and Use of Laboratory Animals published by the US National Institutes of Health (NIH Publication No. 85–23, revised 1996). The experimental protocols were approved by the University of Jilin’s Committee for Ethical Review of Research (Permit Number: 2010–069). All surgery was performed under sodium pentobarbital anesthesia, and all efforts were made to minimize suffering. No specific permissions were required for these experiments and the field studies did not involve endangered or protected species.

### Animal Groups and Diets

Forty male Sprague-Dawley (SD) rats aged 4 weeks and weighing 128.6±2.9 g were purchased from the National Animal Breeding and Research Centre, Beijing, China. The rats were fed a commercial diet (Kangqiao, Inc., Beijing, China) that contained 32% protein, 5% fat, 2% fiber, 1.8% Ca, 1.2% P and 59% N-free extract for 1 week. After this adaptation period, the rats were randomly selected and assigned to 4 groups of 10 rats each. The initial average body weights were similar among the 4 groups. The 4 groups were assigned diets according to the following regimen: (1) control group, high-cholesterol diet; (2) LA15 group, high-cholesterol diet+*L. acidophilus* LA15; (3) B23 group, high-cholesterol diet+*L. plantarum* B23; (4) D17 group, high-cholesterol diet+*L. kefiri* D17. The high-cholesterol diet contained 1% (w/w) cholesterol, 10% lard, 10% sucrose, 0.3% sodium cholate, 0.2% propylthiouracil and a commercial diet mix (Aoboxing Biotech Co., Ltd., Beijing, China). All rats were housed individually in metal cages under a controlled room temperature (23±2°C) and humidity (55±5%) and maintained on a 12 h light-dark cycle. The rats had free access to water and their group-specific diet. Each day during the 4-week study period, the LA15 group, B23 group and D17 group received 2 ml (10^9^CFU/ml) of *L. acidophilus* LA15, *L. plantarum* B23 or *L. kefiri* D17, respectively, intragastrically. The control group received an equivalent amount of normal saline. Body weights were recorded weekly, and food consumption was monitored daily. After 28 days of gavage, the rats were fasted for 12 h and euthanized. The weight of the livers was measured.

### Assay for Serum Lipid Measurement

The overnight fasted rats were bled from the tail vein under sodium pentobarbital anesthesia on the 28th day of the study period. Approximately 1 ml of blood was taken from each rat, transferred to nonheparinized vacuum collection tubes and kept on ice for 30 min. The tubes were then centrifuged at 2,000×g for 20 min at 4°C. Serum total cholesterol (TC), high-density lipoprotein cholesterol (HDL-C), low-density lipoprotein cholesterol (LDL-C) and triglycerides (TG) were measured with commercial kits (Biosino Biotechnology and Science, Beijing, China).

### Assays for Liver and Fecal Cholesterol

After euthanasia, the rat livers were removed, rinsed with a physiological saline solution, blotted dry and weighed. After liver and fecal samples were extracted with a chloroform-methanol (2∶1) solvent, the liver and fecal cholesterol concentrations were determined using the same kits used to determine the serum cholesterol levels [48]. Fecal cholic acid levels were measured using published methods [49].

### Microbial Analysis

Fecal samples were collected on days 0, 28 and 42 (2 weeks after stopping *Lactobacillus* administration). At each sampling, microbial analysis and reisolation of the strains were performed as follows: fecal samples were suspended (1∶10 w/v) in physiological solution and tenfold serially diluted, and 100 µl of the appropriate dilution was plated onto Rogosa agar (Oxoid) with or without 26 µg/ml vancomycin (Sigma-Aldrich, Missouri, USA). The vancomycin-resistant lactobacilli were enumerated on Rogosa-tetracycline agar. The plates were incubated anaerobically for 3 d at 37°C. Ten to twenty percent of the total colonies, randomly selected from countable Rogosa tetracycline agar plates, were isolated and checked for purity. Phenotypic identification of the LAB was achieved using the API 50CHL kit (BioMérieux) based 16S rRNA similarity and by DNA homology, as determined fluorometrically by the method of Ezaki et al. [50]. Total anaerobe counts were obtained using CDC anaerobe blood agar [51], and the plates were incubated anaerobically for 3 d at 37°C. For coliform counts, a single aliquot (1 ml) of each dilution was spread on Petrifilm™ *E. coli*/Coliform Count Plates (3M Corporation, St Paul, Minnesota). The plates were incubated at 37°C for 2 d. Colonies were identified and counted in accordance with the manufacturer’s instructions.

At different times following gavage with the *Lactobacillus* strains (days 0, 28 and 42), rats were sacrificed by Ketamine-HCl anesthesia. The small intestine and colon were removed aseptically and 1 gram of each sample was transferred to a tube with 9 ml of 0.9% NaCl solution and homogenized by vortexing for 10 mins before dilution and cultivation on Rogosa agar (Oxoid). Isolation and identification of the colonies were performed according to standard microbiological methods, as described above.

### Statistical Analysis

Data analysis was carried out with SPSS, Inc. software (version 10.0). One-way ANOVA was used to study any significant difference between means with a significant level of *P*<0.05. Critical difference values were used to perform multiple comparisons between means. All data are expressed as the mean ± standard deviation.
